# Cervicofacial necrotizing fasciitis among patients attending the Muhimbili National Hospital, Dar es Salaam, Tanzania

**DOI:** 10.1186/s12879-019-4267-x

**Published:** 2019-07-19

**Authors:** Arnold A. Mtenga, Boniphace M. Kalyanyama, Sira S. Owibingire, Karpal S. Sohal, Elison N.M. Simon

**Affiliations:** 1 Department of Dental Services, Muhimbili National Hospital, Dar es Salaam, Tanzania; 2 Department of oral and maxillofacial surgery, Muhimbili University of Health and Allied Sciences, P.O. Box 65014, Dar es Salaam, Tanzania

**Keywords:** Cervicofacial, Necrotizing fasciitis, Tanzania

## Abstract

**Background:**

Cervicofacial necrotizing fasciitis (NF) is a rare life-threatening infection in the head and neck region that characteristically spreads along the fascial planes to involve subcutaneous tissues, fascia and fat, however, in late stages it can involve muscles and skin. The aim of this study was to determine the occurrence of cervicofacial NF among patients attending treatment at the Muhimbili National Hospital (MNH).

**Methods:**

This was a prospective descriptive cross-sectional hospital-based study which was carried at Muhimbili National Hospital (MNH) from May 2013 to April 2014. It included 42 patients with cervicofacial NF. They were interviewed for demographic information, chief complaints, symptoms, duration and treatment received before reporting at MNH. A thorough assessment of general health condition of the patients and laboratory investigations were followed by management according to MNH protocol. Data obtained from these patients were analyzed using Statistical Package for Social Sciences SPSS 20.

**Results:**

During the study period, 151 patients reported at MNH with odontogenic infections. A total of 42 (27.8%) patients satisfied our diagnostic criteria for cervicofacial NF. The age range was 15 years to 83 years (mean 43.95, SD +/− 16.16). Greater (35.7%) proportion was in the age group of 30–39 years with 31 (73.8%) males and 11 (27.2%) females making a male to female ratio of 2.8:1.

Fifteen (35.7%) patients had at least one co-existing systemic condition, which included anaemia in 5 (11.9%) patients, followed by diabetes mellitus (DM) and malnutrition 4 (9.5%) patients each and HIV infection 2 (4.8%) patients. Others were combination of; HIV infection and malnutrition, HIV infection and anaemia and diabetes mellitus and anaemia each in one (2.4%) patient. There was a mortality of 42.9% comprising of 14 (33.3%) males and 4 (9.6%) females.

**Conclusions:**

Cervicofacial NF is a polymicrobial infection, requiring surgery, antibiotics and management of co-existing systemic conditions. Anaemia, diabetes mellitus and malnutrition were the main co-existing systemic conditions. The rather high mortality was mainly attributable to late reporting.

## Background

Necrotizing fasciitis (NF) is a rare but life-threatening polymicrobial soft tissue infection characterized by rapid and progressive necrotizing process of the subcutaneous tissues, fats and fascial planes, with resulting skin gangrene and systemic toxicity [1–8]. It commonly affects the extremities, abdominal wall and perineum following surgery or trauma, particularly in individuals with underlying systemic diseases such as diabetes mellitus, arteriosclerosis, chronic renal failure or malnutrition [3–6]. Cervicofacial region involvement is less than 10% and usually the precipitating factor is an odontogenic infection [9, 10].

Panda et al. (2004) reported that almost 70% of patients with NF had an underlying systemic condition that included anaemia, diabetes mellitus, pulmonary diseases, chronic renal failure, alcoholic addiction, HbsAg positive serology and Arnold Chiari malformation [10]. Case reports of cervicofacial infection of odontogenic origin are available in the literature [11–13]. This type has a rapidly progressive course and, if left untreated, is associated with a mortality of between 22 and 100% [1, 5, 9, 14].

Several prognostic factors have been identified to influence survival in NF. These factors include delay in surgical intervention, development of mediastinitis, and the presence of medical co-morbidities [1]. Others are old age (especially age > 60 years), female sex and delayed referral [1, 10]. Meticulous surgical debridement, supplemented by appropriate antibiotic coverage, hyperbaric oxygen therapy and meticulous daily wound dressing have been advocated and accepted as treatment of NF [15, 16].

Most of the available literature on cervicofacial NF is largely based on western countries where delay in seeking medical care, lack of personnel, self-prescription and poor nutrition are not common problems. Except for a few [4, 17, 18], the existing literature consists almost entirely of individual case reports and several small case series, which do not adequately permit reporting of patterns of presentation and management.

Therefore, the aim of this study was to determine the occurrence, predisposing factors and outcome of treatment of cervicofacial NF among patients attending treatment at the Muhimbili National Hospital (MNH), Dar es Salaam, Tanzania.

## Methods

This study took place at the Oral and Maxillofacial unit of the Muhimbili National Hospital (MNH). It was a prospective descriptive cross-sectional hospital-based study that took place in 2013/14.

All patients who attended treatment at the Oral and Maxillofacial unit diagnosed with orofacial infections were included after voluntarily consenting to participate in this study. Orofacial infection was considered when the infection was found to originate from the tooth or supporting structures. Cervicofacial necrotizing fasciitis was diagnosed based on the presence of fascial necrosis, undermining of skin, gangrenous tissue, primary involvement of the face and neck.

Participants were interviewed using a prepared questionnaire whereby data on the demographic pattern, chief complaints, duration of chief complaints and clinical presentations were collected. Confidentiality was maintained and identification numbers instead of patients’ names were used in the questionnaires and clinical examination forms.

A thorough general, extra and intraoral examination was carried out and the findings on general health status of the patient, dentition involved, and extent of the disease was recorded.

Routine and specific laboratory investigations were carried out to all patients with cervicofacial NF. Haematological investigations were carried out. In patients who random blood sugar tests indicated that they had high blood sugar levels, fasting blood sugar was measured during the three consecutive days. Human Immunodeficiency Virus (HIV) test was done and for those who tested HIV positive CD4 count was obtained. Orthopantomograph and chest x rays were done.

Pus was collected aseptically by aspiration using a 20 cc syringe and was immediately immersed in transport media (Stuart transport media). Within 30 min after collection, the specimens were taken to the microbiology laboratory for culture and sensitivity test. Pus swabs were inoculated onto blood agar, Mac Conkey agar and chocolate agar plates.

Drug susceptibility testing was done by disc diffusion method (Kirby- Bauer’s technique).

All the patients started treatment on the day of reporting to the hospital. Under local anaesthesia incision and drainage and debridement of the necrotic tissues were done. The wounds were irrigated with 3% hydrogen peroxide and washed by normal saline then dressed with a sterile piece of gauze containing metronidazole jelly. For patients who presented with an offending tooth without trismus and their general condition allowed, extraction was performed at the same sitting when the incision and drainage and/or debridement was done.

Initial surgery was followed by a combination of broad-spectrum antibiotics which included ceftriaxone 1 g intravenously (I.V) once daily, metronidazole 500 mg I.V after every 8 h, gentamycin 80 mg (I.V) twice a day were prescribed. I.V fluids were given to hydrate and detoxicate the patients. Subsequently, debridement of the necrotic tissues until fresh bleeding appeared was done after 2nd to 3rd day and wound dressing was performed twice daily. For anaemic patients, blood transfusion was given to raise the hemoglobin levels above 10 g/dl. All patients were provided with a highly nutritious diet.

Data were entered in the computer and analysed using Statistical Package for Social Sciences programme (SPSS) software version 20 [19]. The actual age (in numerical form) was represented as the mean and standard deviation (SD) while the categorical variables were represented as frequencies and percentages. For the purpose of statistical analysis, the age was dichotomized into groups of ≤40 years and above 40 years, and duration of hospitalization was categorized into ≤7 days, 8–21 days and > 21 days. Fisher’s test was used to compute the *p*-value. The p-value for decision of association was set at *p* < 0.05.

## Results

One hundred and fifty-one patients with orofacial infections were seen during the study period. Forty-two (27.8%) patients comprising of 31(73.8%) males and 11(26.2%) females were diagnosed with cervicofacial NF. The age ranged from 15 to 83 years with a mean of 43.95 ± 16.16. The 30–39 years age group was most affected followed by the 60+ and 40–49 years age groups. Males were more affected than females in almost all age groups except the 10–19 years and 60+ years age groups (Table 1).

**Table 1:** Distribution of study patients with cervicofacial NF according to age and sex

Age Group (Years)	Sex		Total	%
	Male	Female		
10–19	0	1	1	2.4
20–29	3	2	5	11.9
30–39	13	2	15	35.7
40–49	7	0	7	16.7
50–59	4	1	5	11.9
60+	4	5	9	21.4
Total	31	11	42	100

Eighteen (42.9%) of the patients had primary school education and 6 (14.3%) had secondary school education while 16 (38.1%) had no formal education. Sixteen (38.1%) patients had no formal occupation, while 10 (23.8%) were peasants and 8 (19.0%) were private employees. Only 3 (7.1%) were civil servants.

During their initial visits to health facilities 19 (45%) patients had toothache due to decayed teeth only while 23 (55%) patients had both decayed teeth and toothache associated with swelling. In 29 (69%) patients the offending teeth were not extracted at the health facilities where they first reported prior to referral to MNH.

**Fig. 1:**
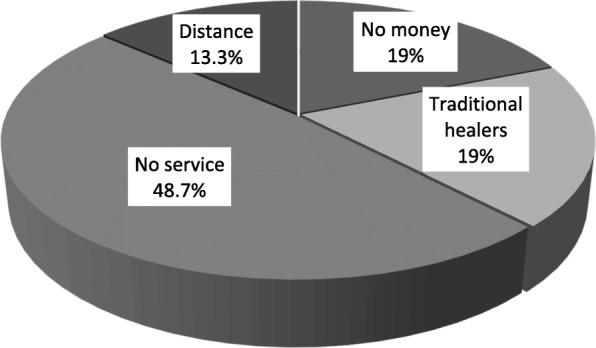
Frequency distribution of factors that were associated with delay in seeking for treatment in patients with cervicofacial NF

Thirty-seven (88.1%) patients reported to health facilities more than a week from the onset of the disease. The majority (48.7%) of the patients delayed seeking dental treatment due to lack of oral health care services in their localities, inability to pay for treatment (19.0%), attending to traditional healers before seeking medical care (19.0%) and long distances from the health facilities (13.3%) (Fig. 1). Only one patient reported within the first three days since the onset of disease.

**Fig. 2:**
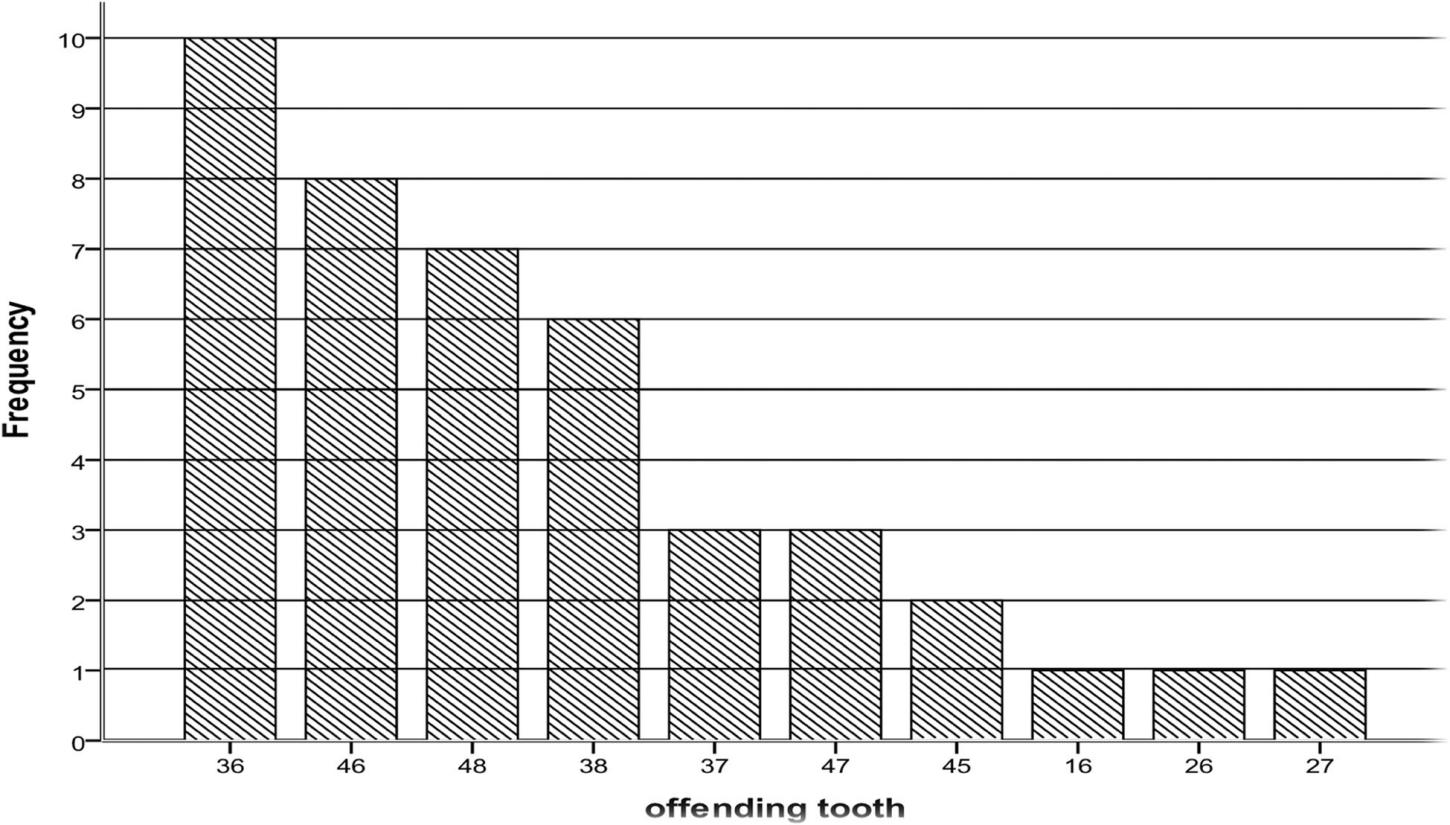
Frequency distribution of the offending teeth in patient with cervicofacial NF

On admission, all patients presented with pain and swelling, 30 (71.4%) patients were clinically dehydrated, 19 (45.2%) presented with difficulty in breathing and 16 (38.1%) had difficulty in swallowing. In 37 (88.1%) patients the lower molars were the associated carious teeth and only 3 (7.1%) patients had a maxillary tooth associated with cervicofacial NF (Fig. 2).

Eighteen (42.9%) patients, had co-existing systemic conditions, of whom 15 (83.3%) had at least one co-existing systemic condition. Anaemia was the most frequently encountered systemic condition (Table 2).

**Table 2:** Distribution of study participants with cervicofacial NF according to co-existing systemic condition and sex

Co-existing systemic condition	Sex		Total
	Male	Female	
Anemia	5 (71%)	2 (29%)	7 (100%)
Diabetes Mellitus	3 (60%)	2 (40%)	5 (100%)
Malnutrition	5 (100%)	–	5 (100%)
HIV + ve	3 (75%)	1 (25%)	4 (100%)

The growth of bacterial colonies was observed in specimens of 30 (71.4%) patients. *S. aureus* was the most commonly isolated micro-organism (Table 3). Drug susceptibility testing showed that the isolates were highly sensitive to ceftriaxone (96.7%), gentamycin (86.7%) and erythromycin (83.3%). Resistance was observed to ampicillin (86.7%) and cloxacillin (90%).

**Table 3:** Bacteriological pattern in patients with cervicofacial NF

Bacteria	Gender		Total	%
	Male	Female		
*S. aureus*	9	3	12	28.6
*S. pyogenes*	7	2	9	21.4
*K. pneumoniae*	4	0	4	9.5
*P. aeruginosa*	2	1	3	7.1
*E. coli*	1	1	2	4.8
*H. influenza type b*	2	0	2	4.8
No growth	6	4	10	23.8
Total	31	11	42	100

Less than a half (42.9%) of the patients healed uneventfully while 3 had to undergo skin grafting and other three patients suffered contractures. There was a mortality of 42.9% comprising of 14 (77.8%) males and 4 (22.2%) females. Mortality was found to be significantly associated with dehydration during admission (*p* = 0.005) and short duration of hospital stay (*p* = 0.000) while it was not significant for the other factors (Table 4). Most of the deaths (12, 66.7%) occurred within the first week of admission, of which 8 (66.7%) occurred within the first four days.

**Table 4:** Distribution of the treatment outcome of patients with cervicofacial NF according to different factors

Associated Factors	OUTCOME		*p*-value
	Died	Survived	
Age			
0–40 years	9 (39.1%)	14 (60.9%)	0.756
41+ years	9 (47.4%)	10 (52.6%)	
Sex			
Male	14 (45.2%)	17 (54.8%)	0.731
Female	4 (36.4%)	7 (63.6%)	
Underlying systemic condition			
Yes	10 (55.6%)	8 (44.4%)	0.211
No	8 (33.3%)	16 (66.7%)	
Dehydrated on admission			
Yes	17 (56.7%)	13 (43.3%)	0.005
No	1 (8.3%)	11 (91.7%)	
Difficulty in breathing during admission			
Yes	10 (52.6%)	9 (47.4%)	0.349
No	8 (34.8%)	15 65.2%)	
Difficulty in swallowing during admission			
Yes	9 (56.3%)	7 (43.8%)	0.210
No	9 (34.6%)	17 (65.4%)	
Duration of Hospital stay			
≤ 7 days	12 (100%)	–	0.000
8–21 days	4 (40%)	6 (60%)	
≥ 22 days	2 (10%)	18 (90%)	

## Discussion

The mean age of 43.49 years as was found in this study did not differ much with findings of other studies [7, 20–22]. It, however, differed from the earlier assertions by Jacob et al. (2006) in Nigeria who reported that over half (57.1%) of the patients were children aged < 15 years and that of Nyako et al. (2006) who reported that in Ghana, cervicofacial NF affected all age groups equally [22, 23].

In this study it was clinically and radiographically proven that in all the cases a dental problem was the primary cause of cervicofacial NF. Incongruency with information in the literature, the findings showed that NF was most commonly associated with the mandibular molars [24–26]. This augments well with reports of epidemiological studies in Tanzania which showed that lower molars were the teeth that were most commonly affected by dental caries [27, 28].

The proportion (42.9%) of patients with co-existing systemic conditions did not differ from other reports [16, 26, 29], however, while in these reports DM was predominant, in this study anaemia dominated. In several other reports anaemia was also found to co-exist with cervicofacial NF [10, 30]. When cervicofacial NF occurs in immunocompromised individuals like in HIV positive patients it progresses rapidly and can be potentially fatal [17, 22]. In this study two out of four patients who were HIV positive died of septicemia and multi-organ failure. Generally, the proportion of those who died of the disease (42.6%) was rather high compared with other reports [5, 14]. Most of the deaths were due to prolonged delay in reporting at MNH, which was evidenced by the state of dehydration at the time of admission and the fact that majority died within four days of admission.

Five (11.9%) patients were malnourished, which might have been a contributing factor to the development of cervicofacial NF. In addition to the underlying malnutrition, difficult or inability to eat during the course of the disease further weakened the patients. This finding is supported by others who also found malnutrition in their patients because of the reduced oral intake [6, 22]. Dietary supplements in the form of a high protein diet are necessary for such patients. From the findings of this study, it is noteworthy, that even in the absence of underlying factors cervicofacial NF may result from unattended dental caries leading to serious infections of odontogenic origin. Although there are sentiments that extraction during acute infection may disseminate the infection, in our situation immediate extraction was considered to minimize pain and stimulate resolution of the infection [31].

So far, in Tanzania, oral health services are only readily available in the district, municipal and regional hospitals. Therefore, in most villages in Tanzania, individuals with dental problems are compelled to travel long distances to the centres with qualified oral health personnel. The financial implications to these usually economically deprived individuals contribute greatly to the tendency to overstay with unattended disease [22]. In the absence of universal health insurance to the majority of patients as is the case in Tanzania, the whole burden of health care costs is solely borne by the patients. Like in other African countries, possibly this leads to many patients (19% in this study) consulting traditional healers who happen to be within their reach [32]. The fact that majority of the patients in this study group had low level of education or did not have any formal occupation might have compounded the situation.

The present study revealed that there is a long existing myth among both lay people and some health personnel (including oral health personnel) that a tooth should not be extracted in the presence of swelling resulting from infection. This led to several of the patients who attended treatment at different health facilities, including district and regional hospitals, getting prescriptions for antibiotics only in the belief that the swellings would later improve or resolve and allow tooth extraction. To the contrary, such swellings worsened and led to eventual progression to cervicofacial NF. Delay in seeking appropriate oral health care is therefore multifactorial and contributed to the advanced stages of disease that patients presented with. A similar situation has been reported in New Zealand, where it was found that patients with cervicofacial NF delayed seeking treatment due to limited health knowledge; and lack of access to health care or barriers to that access [33].

Similar to available information polymicrobial infection was the most common aetiology of NF in this group of patients [21, 28, 29, 34]. Delay in instituting appropriate measures allows the infection to spread in any direction, often ascending to the temporal space or descending to the chest wall and resulting in mediastinitis both of which are often fatal [1, 11, 12, 34].

The commonest isolated organism was *S. aureus* which is similar to what has been reported in previous studies [10, 21, 22]. The microbial susceptibility pattern in the present study showed that the microorganisms were very sensitive to ceftriaxone, erythromycin and gentamycin, which is consistent with other findings at MNH [27, 35]. These studies also showed that resistance to ampicillin and cloxacillin was very high. Due to this polymicrobial nature of cervicofacial NF, patients should initially be managed empirically by high dose of broad-spectrum antibiotics while waiting for culture and sensitivity results. Similar to the experiences of others as displayed in different reports the significant role of surgical debridement in the management of CFNF is underscored [33, 35]. In this study, debridement was carried out under local anaesthesia on every 2nd to 3rd day. Although general anaesthesia (GA) would have been more ideal in treating these patients, in our setting there is limited theatre space and a good proportion of the patients were very weak on reporting that they would require initial stabilization before they would be fit for GA. Nevertheless, given the urgency of dealing with NF and the required frequency of performing debridement, meticulous care was taken to make sure that debridement under local anaesthesia was sufficiently effected in all patients.

There are some inherent limitations in this study. The study was conducted in a single institute; thus the results might not give the true incidence of CFNF in the region. Moreover, majority of the patient had received antibiotics prior to attending treatment in our center, and this may have affected culture and sensitivity results. Despite these limitations, the findings of this study give useful information about aetiology, risk factors and outcome of CFNF.

## Conclusion

Cervicofacial NF affected all age groups above 10 years with the highest frequency in the 30–39 years age group. Anaemia, diabetes mellitus and malnutrition were the main co-existing systemic conditions. Cervicofacial NF is a polymicrobial infection that required a combination of antibiotics, surgical measures and management of co-existing systemic conditions to control it. The rather high mortality in this group of patients was mainly attributable to late reporting for appropriate care.

### Recommendations

Continuing education to health personnel with an emphasis on appropriate handling of patients presenting with NF is encouraged.

Early institution of broad-spectrum antibiotic therapy, local surgical debridement and management of the underlying systemic conditions is of utmost importance.

Further long term multicenter studies on cervicofacial NF in Tanzania are necessary.

## Data Availability

The complete set of data and materials used in this study are freely available from the corresponding author on reasonable request.

## Abbreviations

CCP: Comprehensive Chemistry Panel
CD4: Cluster of Differentiation 4
ESR: Erythrocyte Sedimentation Rate
FBP: Full Blood Picture/count
HIV: Human Immunodeficiency Virus
MNH: Muhimbili National Hospital
MUHAS: Muhimbili University of Health and Allied Sciences
NF: Necrotising fasciitis
